# Genital Epstein Barr Virus is associated with higher prevalence and persistence of anal human papillomavirus in HIV-infected men on antiretroviral therapy

**DOI:** 10.1186/s12879-016-1356-y

**Published:** 2016-01-25

**Authors:** Sara Gianella, Christine C. Ginocchio, Eric S. Daar, Michael P. Dube, Sheldon R. Morris

**Affiliations:** 1 University of California San Diego, 500 Gilman Drive MC 0679, La Jolla, CA 92093-0679 USA; 2 North Shore-LIJ Health System, Lake Success, NY USA; 3 Hofstra North Shore-LIJ School of Medicine, Hempstead, NY USA; 4 Los Angeles Biomedical Research Institute at Harbor-UCLA Medical Center, Torrance, CA USA; 5 University of Southern California Keck School of Medicine, 1300 N. Mission Road, Suite 349, Los Angeles, CA 91106 CA USA; 6 University of California San Diego, 200 Arbor Dr., Mail code 8208, San Diego, CA 92103 USA

## Abstract

**Background:**

Epstein Barr virus (EBV) and human papillomavirus (HPV) can co-exist in pharyngeal and cervical malignancies. However, the natural history and factors associated with persistent HPV infection among HIV-infected men who have sex with men (MSM) are unclear.

**Methods:**

131 HIV-infected MSM were followed for 48 weeks and screened for multiple co-infections, including seminal EBV DNA and high risk (HR)-HPV messenger RNA (mRNA) at several sites (semen, anal, pharynx). Primary analysis tested if seminal EBV shedding was associated with increased prevalence of HR-HPV at baseline using univariate tests and multivariable logistic regression. In participants with detectable anal HR-HPV at baseline, we tested if presence of seminal EBV shedding at baseline was also predictive of reduced HR-HPV clearance by log-rank test (over 48 weeks of follow-up).

**Results:**

Baseline prevalence of HR-HPV was: anal 44 % (*N* = 54/121); pharynx 3.8 % (*N* = 5/131); semen 7.1 % (*N* = 7/98). Seminal EBV shedding was present in 28 % of participants and was associated with more than double the prevalence of detectable anal HR-HPV mRNA (71.4 % for EBV shedders versus 33.3 % for non-shedders, *p* < 0.01). In participants with detectable anal HR-HPV at baseline, we found increased persistence of HR-HPV over 48 weeks of follow-up (measured as time to first negative HR-HPV test in the EBV shedding group (*p* < 0.01).

**Conclusions:**

Seminal EBV shedding was associated with an increased risk of having detectable anal HR-HPV in a cohort of HIV-infected MSM on suppressive ART. Future studies should examine if co-infection with EBV and HR-HPV may act synergistically in pathogenesis of anal cancer in HIV-infected individuals.

## Background

Human Papillomaviruses (HPV) are sexually transmitted and can infect the genital and anal areas, mouth, and throat [1]. Over 100 types of HPV are reported and at least 14 are considered high-risk (HR) for leading to cancer of infected body sites [2, 3]. While the association between HR-HPV infection and cervical cancer in women is universally recognized, convincing evidence also demonstrates a strong link between HR-HPV infection and anal cancer especially among men who have sex with men (MSM) [4, 5]. The incidence of anal cancer in the MSM population has risen in the last few decades, and HIV-infected MSM are at the highest risk with approximately 80 per 100,000 men (compared to 2 per 100,000 men in the general population), even with antiretroviral therapy (ART) [6]. The global estimate of HR-HPV infection in MSM in the US ranges from 48 to over 95 % for the HIV-infected population [1, 3, 7, 8]. Although many HR-HPV infections in men and women have been shown to be transient in nature, a small percentage persist and can progress to genital warts, pre-neoplastic and malignant lesions of the anus, penis, and oropharynx [5]. The natural history of HR-HPV infection and what factors are associated with persistent HR-HPV infection in HIV-infected MSM is still unclear.

Another common viral infection found in MSM and in the general population is Epstein Barr virus (EBV), which is the cause of infectious mononucleosis [9]. After primary infection, EBV establishes a latent infection and can cause episodic bursts of replication particularly in the genital and oral mucosa [10]. Interestingly, latent infection with EBV can act as a carcinogenic co-factor in several epithelial cell malignancies [11], and EBV and HR-HPV can co-occur in pharyngeal and cervical malignancies [12–14]. Presence of EBV co-infection is associated with a five-fold higher risk of integration of concurrent HR-HPV into the human genome [13], which is an important step in the progression to invasive carcinoma. In this study, we performed a post-hoc analysis to investigate if presence of active EBV replication (as measured in the seminal plasma) was associated with increased prevalence and reduced clearance of concurrent HR-HPV infection in the anal, pharynx and genital mucosa of HIV-infected MSM during suppressive ART.

## Methods

### Participants, samples and clinical laboratory tests

The studies were conducted with appropriate written consent and were approved by the Human Research Protections Program at the University of California San Diego, Los Angeles Biomedical Research Institute at Harbor-UCLA Medical Center, the University of Southern California and the BioMedical Research Alliance of New York.

A total of 179 participants were prospectively enrolled and followed as part of the California Collaborative Treatment Group (CCTG) 592 study, which was an internet-based behavioral intervention study of HIV-infected MSM at high-risk for sexually transmitted infections. At baseline, 131 participants were receiving ART with HIV RNA <500 copies/ml in blood plasma, and were included in this analysis. As part of the study protocol, blood and semen samples were collected at baseline [15], and anal and pharyngeal swabs were collected at baseline and every 12 weeks for a total of 48 weeks. Anal and pharyngeal specimens were collected using the Aptima collection kits and stored at room temperature for immediate shipping. At every visit, subjects completed self-reported questionnaires for sexual risk behavior, drug use and adherence to ART in the previous 4 weeks. Blood CD4+ T-lymphocyte subsets were measured by flow-cytometry (CLIA certified local laboratories), and HIV RNA levels in blood plasma were quantified by the Amplicor HIV Monitor Test (Roche Molecular Systems Inc.).

### Quantification of viral nucleic acid levels from multiple mucosal sites

Real-time PCR was used to measure levels of HIV RNA and different human herpesviruses (HHV) in semen at baseline (cytomegalovirus EBV, herpes simplex viruses [HSV] types 1 and 2, and HHV types 6, 7, and 8) [15].

Levels of mRNA from the E6/E7 oncogenes for 14 HR-HPV types (16/18/31/33/35/39/45/51/52/56/58/59/66/68) were measured by Aptima HPV Assay (Hologic Inc., San Diego CA) in semen (at baseline), anus and pharynx (baseline and every 12 weeks). Additionally, Aptima HPV 16 18/45 Gentoyping Assay was run on a subset of anal (*N* = 52) and pharyngeal (*N* = 5) samples tested positive for HR-HPV.

### Statistics

Statistical analyses were performed using SAS (version 9.2). For this post-hoc analysis, viral load variables were transformed to logarithm-base ten values. The primary analysis tested if the presence of detectable genital EBV at baseline was associated with increased prevalence of HR-HPV infection (at baseline) and reduced clearance of HR-HPV DNA over the following 48 weeks. Continuous variables were tested for normality with the Shapiro-Wilk test, and if not normally distributed, comparisons were performed using nonparametric tests or values were dichotomized. Comparisons between groups (HR-HPV shedding status, seminal EBV shedding status) were performed using Fisher-exact test (for sparse categorical variable), *t*-test (for continuous, normally distributed variables) or Mann Whitney *U* test (for continuously, non-normally distributed variables). Test of correlation between continuous variables was done with Spearman rank correlation. For the outcome of HR-HPV clearance, we used log-rank test for time to first and second consecutive negative HPV test. The association of EBV and HR-HPV prevalence was analyzed by logistic regression including any factor that was independently associated with HPV considering behavioral factors (number of sex partners and anal sex acts, use of methamphetamine and other drugs), blood and seminal plasma HIV levels, current and nadir CD4 T and CD8 T cell count, and genital shedding of other HHV.

## Results

### Study participants’ demographics and clinical data

The majority of study participants (84.0 %) had <50 HIV RNA copies/ml in blood plasma. The median CD4 T cell count was 604 cells/μL (IQR: 414–761). Characteristics of this cohort have been previously described [15,16], and are summarized in Table 1.

**Table 1:** Demographics and co-infections at baseline

Characteristics		
MSM, n (%)		131 (100)
Age (years), median (IQR)		47 (39–52)
Race, n (%)	White	84 (64.1)
	Black	41 (31.3)
	Other	6 (4.6)
Hispanic Ethnicity, (n, %)		43 (32.8)
HIV RNA <50 copies/ml, n (%)		110 (84.0)
≥90 % ART adherence past 4 weeks, n (%)		115 (87.8)
CD4+ T-cell counts/μL, median (IQR)		604 (414–761)
Unprotected anal sex acts past 4 weeks, median (IQR)		0 (0–2)
Any drug use, n (%)		46 (35.9)
Median Number Male Sexual Partners past 4 weeks, median (IQR)		3 (1–6)
High Risk HPV mRNA at baseline, n (%)		
Semen (*n* = 98)		7 (7.1)
Anal (*n* = 120)		54 (45.0)
Throat (*n* = 127)		5 (3.9)
Herpeviruses Shedding, n (%)		
Any detectable HHV DNA		83 (63.7)
Any detectable HSV (1 or 2) DNA		3 (2.3)
Any detectable CMV DNA		69 (52.7)
Any detectable EBV DNA		36 (27.5)
Any detectable HHV-6 DNA		9 (6.9)
Any detectable HHV-7 DNA		10 (8.8)
Any detectable HHV-8 DNA		3 (2.3)

Legend: *n* (%) number (percentage) of participants, *MSM* men who have sex with men, *HIV* Human immunodeficiency virus, *ART* antiretroviral therapy, *IQR* interquartile range, *HHV* Human Herpesviruses, *CMV* cytomegalovirus, *EBV* Epstein-Barr virus, *HSV*-*1* and -*2* Herpes simplex virus type 1 and type 2, *HHV-6/-7/-8* human herpes virus type 6/type 7/type 8

### Prevalence of HR-HPV infection at baseline and during 48 weeks follow-up

At baseline, 61 (46.6 %) individuals had detectable HR-HPV mRNA at any site for at least one of the 14 HR genotypes tested. HR-HPV mRNA was most commonly detected from anal swabs with a baseline prevalence of 45.0 % (N = 54/120 evaluable swabs). Using the HR-HPV genotype specific assay on anal mucosa samples (for HPV 16 and 18/45), we observed that HPV 16 was detected more frequently than genotypes 18/45 (i.e., 30.8 % [16/52] compared to 15.4 % [8/52]). Overall, at baseline HR-HPV mRNA was not frequently detected in the pharynx (3.9 % [N = 5/127]), or in seminal secretion (7.1 % [N = 7/98]). None of the 5 HR-HPV positive samples collected from the pharyngeal mucosa were positive for 16 or 18/45 genotypes. Presence of HR-HPV in semen and pharynx was not associated with anal HR-HPV and only 2 individuals had detectable HR-HPV at multiple concurrent mucosal sites. Including all analyzed longitudinal anal swabs for all individuals over 48 weeks of follow-up we found that 85/127 subjects (66.9 %) presented detectable anal HR-HPV and 12/131 (9.2 %) presented detectable pharyngeal HR-HPV (semen samples were only taken at baseline).

### Prevalence of genital herpesvirus replication at baseline

At baseline, 84 (64.1 %) had detectable HHV DNA in their seminal plasma including: EBV (27.5 %), HSV-1/2 (2.3 %), CMV (51.9 %), HHV-6 (6.9 %), HHV-7 (8.4 %), HHV-8 (3.1 %) [15] (Table 1).

### Predictors of detectable HR-HPV mRNA at baseline

**Fig. 1:**
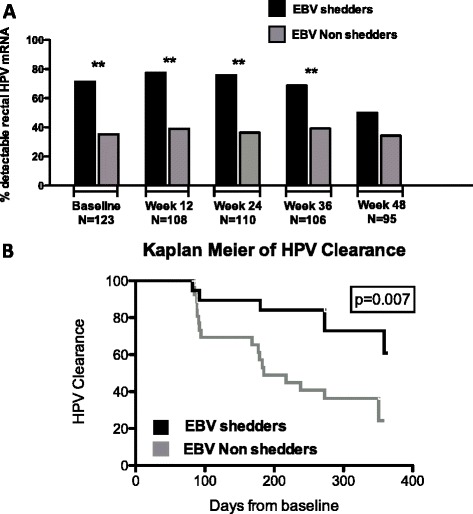
**a** Prevalence of detectable anal HR-HPV mRNA at every study visit (baseline and weeks 12, 24, 36, and 48) divided by groups (EBV shedders versus EBV non-shedders). Black bars show EBV shedders; gray bars show EBV non-shedders. **P < 0.001. **b** Kaplan Meier analysis of time to first negative anal HR-HPV Results among participants with detectable HR-HPV mRNA at Baseline during 48 weeks of follow-up in subjects with detectable seminal EBV DNA (in black) and without detectable seminal EBV DNA (in gray) at baseline

We first investigated possible predictors associated with detectable HR-HPV at baseline (summarized in Table 2). In our post-hoc analysis we found that having seminal shedding of EBV was associated with increased prevalence of detectable anal HR-HPV mRNA compared to no detectable EBV (71.4 % versus 34.1 %, *p* = 0.0002, Fig. 1a). The only other variable associated with increased HR-HPV at the univariate level was lower CD4 T cell count (*P* = 0.01). Among participants with detectable anal HR-HPV (any genotype) there was no statistical difference for genotypes 16 and 18/45 between EBV shedders and non- shedders. Also, having detectable seminal EBV was not associated with significantly increased HR-HPV infection in pharynx (2.9 % versus 4.2 %, *p* = 1.00), and in semen (11.5 % versus 5.6 %, *p* = 0.38) compared to undetectable EBV. Notably, the increased risk of HR-HPV infection associated with EBV presented a similar effect size in semen compared to the anal but this was not statistically significant (likely because of the smaller sample size). None of the other factors (except for detectable EBV DNA and lower CD4^+^ T cells count) was associated with increased HR-HPV mRNA detection. The association of EBV shedding with anal HR-HPV had an adjusted odd ratio of 3.99 (1.62-9.81) when including CD4^+^ count in the model (see Table 2).

**Table 2:** Factors Associated with Prevalent High Risk Anal HPV (Baseline)

Factor	HR HPV+	HR HPV-	OR	*P*-value	Adjusted OR
	(*N* = 54)	(*N* = 66)			(95 % CI)
Any detectable semen EBV DNA, n (%)	25 (46.3)	10 (15.2)	4.8	**<0.01**	3.99 (1.62–9.81)
No detectable semen EBV DNA, n (%)	29 (53.7)	56 (84.9)			
Age (years), mean (95 % CI)	43.8 (40.7–46.9)	46.9 (44.6–49.2)	0.97	0.11	
Caucasian/Non-Hispanic, n (%)	20 (37.0)	18 (27.3)	1.57	0.32	
CD4+ T-cells/μL, mean (95 % CI)	518 (451–586)	631 (572–690)	0.8	**0.01**	0.89 (0.76–1.06)
Blood HIV RNA <50 copies/ml, n (%)	42 (77.8)	58 (87.9)	0.48	0.14	
Any detectable HIV RNA in semen, n (%)	7 (13.2)	6 (9.1)	1.52	0.16	
Any detectable HHV DNA, n (%)	38 (70.4)	40 (60.6)	1.54	0.26	
Any detectable HSV-1/HSV-2 DNA, n (%)	1 (1.9)	2 (3.0)	0.6	0.47	
Any detectable CMV DNA, n (%)	33 (61.1)	32 (48.5)	1.67	1	
Any detectable HHV-6 DNA, n (%)	4 (7.4)	3 (6.0)	1.24	0.17	
Any detectable HHV-7 DNA, n (%)	4 (8.0)	4 (7.6)	1.07	1	
Any detectable HHV-8 DNA, n (%)	2 (3.7)	1 (1.5)	2.5	1	
Number of male partners past 4 weeks (>9), n (%)	7 (17.5)	7 (13.8)	1.33	0.77	
Any unprotected anal sex acts past 4 weeks, n (%)	10 (18.5)	11 (16.9)	1.12	1	
Any Illicit drug use other than marijuana, n (%)	20 (37.7)	21 (32.8)	1.24	0.58	

Legend: *n* (%): number (percentage) of participants, *OR* odds ratio, *IQR* interquartile range, *95 % CI* 95 % confidence intervals, *HIV* Human immunodeficiency virus, *ART* antiretroviral therapy, *HR HPV* high risk human papillomavirus, *HHV* human herpesviruses, *CMV* cytomegalovirus, *EBV* Epstein-Barr virus, *HSV-1* and *-2* Herpes simplex virus type 1 and type 2, *HHV-6/-7/-8* human herpes virus type 6/type 7/type 8, in bold significant p-values (*P* ≤ 0.1). Column under HR HPV+ is for each Factor the N (%) with HR HPV or in continuous the mean (95 % CI) of the Factor in those with HR HPV

### Longitudinal analysis of HR-HPV clearance

Overall prevalence of HR-HPV infection did not change over the course of 48 weeks of follow up (*p* = 0.56, Fig. 1a). Nevertheless, participants with detectable seminal EBV at baseline had higher HR-HPV prevalence at every study visit (*P* < 0.001), except week 48 where sample size was reduced due to loss to follow-up (*P* = 0.15). Among participants with detectable anal HR-HPV at baseline we found increased persistence of HR-HPV in the EBV shedding group over 48 weeks of follow-up (Fig. 1b) measured as time to first and second negative HR-HPV test by log rank test for (*p* = 0.007 and *p* = 0.008).

## Discussion

The prevalence of anal HPV infection in HIV-infected men is high in the US, as is the prevalence of anal infections with multiple HPV types [1, 3]. Findings for clearance of cervical HPV in women cannot be extrapolated to MSM because of anatomic and structural differences, and it is important to understand what factors are associated with increased prevalence and persistence of HPV infection in the HIV-infected MSM population. This post-hoc analysis was performed to investigate the potential interaction of EBV and HR-HPV infections in HIV-infected MSM, based on reports demonstrating co-occurrence of EBV and HR-HPV infection in cervical dysplastic tissue [8, 13, 14]. Additionally, both viruses are carcinogenic and successfully developed immune evasion mechanisms allowing long-term persistence in anatomic tissues [8, 13, 14].

In our cohort of well-characterized HIV-infected MSM, we found a prevalence of 47 % detectable HR-HPV mRNA at baseline. Similar to previous reports, over half of the HR-HPV infections were not genotypes 16 or 18/45, and thus would not be covered by the bivalent and quadrivalent vaccines [3]. The most common site of HR-HPV infection was anal (45 %). However, HR-HPV mRNA was detected at a lower rate also in pharynx (3.9 %) and seminal plasma (7.1 %).

We found that presence of seminal EBV shedding at baseline was the strongest factor associated with detectable baseline HR-HPV infection of anal mucosa. The only other factor associated with detectable HR-HPV mRNA in the univariate analysis was lower CD4^+^ count, but this was not significant after adjusting for presence of EBV. On the other side, the association between EBV shedding and anal HR-HPV had an adjusted odd ratio of 3.99 (1.62–9.81) when including CD4^+^ count in the model (see Table 2).

Differently from previous reports, no association was observed between presence of HR-HPV infection and number of sexual partners, unprotected receptive sex or use of illegal drugs [17, 18].

Among participants with detectable HR-HPV at baseline, we observed that EBV shedding was associated with significantly reduced likelihood of HR-HPV clearing during the following 48 weeks. This is in line with a previous report suggesting that EBV might be associated with higher risk of HR-HPV integration into the human genome [13], which is an important step in the development of invasive carcinoma.

This exploratory study has several limitations. Despite the strong association, our study only included a limited number of HIV-infected men and these results should be confirmed as part of a larger prospective cohort study including both HIV infected and uninfected individuals. Also, the observed association between EBV shedding and HR-HPV infection might be confounded by an unmeasured factor. In particular, there could be a common HIV-related immune defect that could make individuals susceptible to both EBV reactivation and HR-HPV infection. However, it is important to note, that none of the other measured HHV was associated with presence of detectable HR-HPV. Also, the effect of EBV shedding at baseline did diminish over time and by 48 weeks the prevalence of HR-HPV at the anal site was no longer statistically significant between groups. Since EBV seminal replication is intermittent, future studies should collect longitudinal samples to investigate if this observed effect is sustained over time. We also recognize that, since HR-HPV mRNA was detected in anal mucosa, local EBV shedding in seminal plasma may not be directly associated with increased HR-HPV acquisition but be a proxy of EBV replication in the surrounding ano-genital area. Future studies should measure EBV DNA in several anatomic locations to support our observation.

## Conclusions

In summary, we found that EBV shedding was associated with almost double prevalence of anal HR-HPV infection compared to non EBV-shedding. Further investigation is needed to confirm this observation and explore the causal pathways of how these two viruses might interact by evading immune responses and whether this interaction extends to carcinogenesis.

## Abbreviations

ART: antiretroviral therapy
CCTG: California Collaborative Treatment Group
CMV: cytomegalovirus
EBV: Epstein Barr virus
HHV: human herpesviruses
HPV: human papillomavirus
(HR)-HPV: high risk human papillomavirus
HSV: herpes simplex virus
IQR: interquartile range
MSM: Men who have sex with m
(m)RNA: messenger RNA
